# Supplementary material for: Fabrication of AuNPs/MWCNTS/Chitosan Nanocomposite for the Electrochemical Aptasensing of Cadmium in Water
Source: Sensors (Basel). 2021 Dec 24;22(1):105. doi: 10.3390/s22010105 (PMC8747752; doi:10.3390/s22010105)
Supplement: Supplementary file 1 [file sensors-22-00105-s001.zip › sensors-1437706-supplementary.pdf]

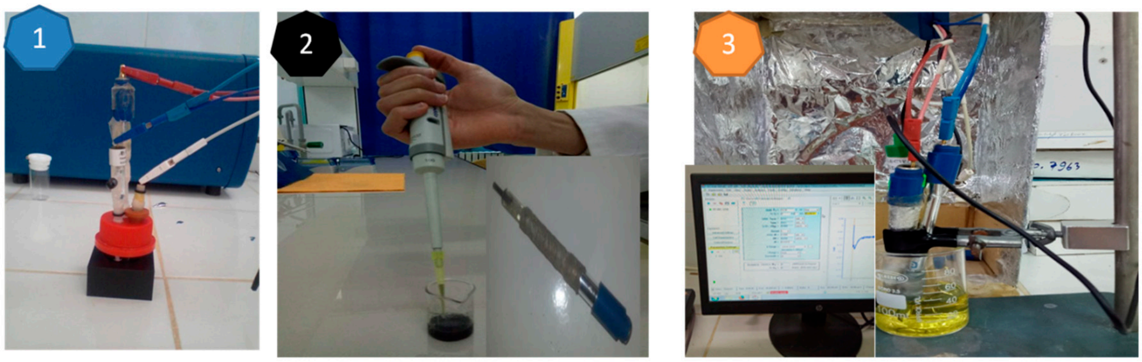

1/ Working electrode: *glassy carbon electrode (GCE), counter electrode: platinum wire ,*
Reference electrode: saturated electrode of calomel (SCE).
2/ Electrode modification with a drop of 6 µL of 0.5 mg/ ml CNTs–CS solution
3/Electrodeposition of AuNPs on CNTs–CS –GCE for 400 s at –0.2 V in a 0.2 M Na_2SO_4 *solution containing 1 mM* $HAuCl_4$

Figure S1. Pictures of the real sensor and some fabrication steps.
